# A Fast Multiobjective Optimization Strategy for Single-Axis Electromagnetic MOEMS Micromirrors

**DOI:** 10.3390/mi9010002

**Published:** 2017-12-23

**Authors:** Francesco Pieri, Alessandro Cilea

**Affiliations:** Department of Information Engineering, Università di Pisa, 56122 Pisa, Italy; acilea@maflex.it

**Keywords:** Micro-electro-mechanical systems (MEMS), MOEMS, micromirrors, multiobjective optimization, pico-projectors, magnetic actuation

## Abstract

Micro-opto-electro-mechanical (MOEMS) micromirrors are an enabling technology for mobile image projectors (pico-projectors). Low size and low power are the crucial pico-projector constraints. In this work, we present a fast method for the optimization of a silicon single-axis electromagnetic torsional micromirror. In this device, external permanent magnets provide the required magnetic field, and the actuation torque is generated on a rectangular multi-loop coil microfabricated on the mirror plate. Multiple constraints link the required current through the coil, its area occupancy, the operating frequency, mirror suspension length, and magnets size. With only rather general assumptions about the magnetic field distribution and mechanical behavior, we show that a fully analytical description of the mirror electromagnetic and mechanical behavior is possible, so that the optimization targets (the assembly size, comprising the mirror and magnets, and the actuation current) can be expressed as closed functions of the design parameters. Standard multiobjective optimization algorithms can then be used for extremely fast evaluation of the trade-offs among the various optimization targets and exploration of the Pareto frontier. The error caused by model assumptions are estimated by Finite Element Method (FEM) simulations to be below a few percent points from the exact solution.

## 1. Introduction

Micro-opto-electro-mechanical (MOEMS) mirrors have a long history, with the first prototypes used in image projection dating 1975 [1]. Successful applications, however (with the important exception of the celebrated, TI-developed Digital Micromirror Device or DMD [2]), have been limited. At the turn of the century, a burst of interest in MOEMS-based optical cross-connect [3] was killed by the dot-com collapse. Today, a renewed interest in micro-mirrors is driven by their envisioned use of MOEMS optical scanners, mainly for two specific applications: LiDARs (Light Detection and Ranging) for autonomous vehicles [4,5,6], and image projectors for mobile devices (pico-projectors) [7,8,9].

Pico-projectors (but also LiDARs) require 2D raster scanning of their field, with a frame rate of the order of tens of Hz [6]. In pico-projectors, scanning along the two directions can be obtained with two different mirrors, a “fast” resonant one for the horizontal (line) scan, and a “slow” one for the vertical scan, but solutions with a single, two-axis mirrors are possible. Micro-electro-mechanical (MEMS) mirrors with electrostatic [10,11], magnetic [12,13], or piezoelectric [14] actuations have all been pursued. Electrostatic actuation, though technologically well-established, requires high driving voltages. The opposite is true for magnetic actuation, were low voltages (but high currents) are typically required. In piezoelectric mirrors, the issues are the integration of the piezo material in the fabrication and the relatively small deflections typically attained with piezoelectric actuators. Holmstrom et al. presented a comprehensive review of MOEMS-based scanners in [15].

Because of their devised use in mobile systems, the design of MOEMS mirrors for pico-projectors poses severe constraints in terms of size and power consumption, issues that are not uncommon in MEMS design in general. Consequently, structured, Computer-Aided-Design (CAD) assisted approaches to MEMS/MOEMS design optimization would be welcome. There is, however, evidence that MEMS design is still largely based on inherently suboptimal ad-hoc, trial-and-error methods [16,17,18,19]. Part of this problem can be attributed to the lower maturity of the MEMS field with respect to other fields of engineering, which also reflects in a lack of efficient integrated tools for optimal design. In the past, because of the multi-domain nature of MEMS, most of the effort in the development of MEMS CAD has been dedicated to FEM simulation tools, of which several exist today. However, the application of optimization methods has received a progressively growing attention since the origin of the MEMS field. In this context, the application of Multiobjective Optimization (MO) is of significant interest.

MO is a systematic approach for the design of any system where the simultaneous minimization (or maximization) of two or more performance metrics (or objective functions in the MO parlance) is desirable, that is, for every but the most trivial design problems. As such, MO is of enormous importance in engineering. In the naïve (though commonly pursued) approach, multiobjective optimization problems are often transformed (more or less deliberately) in the minimization of a single objective, chosen based on the relative importance that the designer assigns to each one, with the other objectives set at reasonable compromise values. This approach is typically repeated in a trial-and-error fashion, until an acceptable solution is reached. MO addresses this kind of problem in a systematic way.

Formally, if the design of a system depends on P variables $x_{1}\ldots x_{P}$, and K performance metrics $f_{1}\ldots f_{K}$ are to be minimized, a MO problem can be stated as [20]

(1) $$\{\begin{array}{ll} \mathrm{minimize}f_{k}\left(x\right),\; & k=1,\ldots ,K\\ \mathrm{constrained}\;\mathrm{to} & \\ h_{m}\left(x\right)=0, & m=1,\ldots ,M\\ g_{n}\left(x\right)\geq 0, & n=1,\ldots ,N\\ x_{pL}\leq x_{p}\leq x_{pH}, & p=1,\ldots ,P \end{array}$$

where $x=\left[x_{1}\ldots x_{P}\right]$ is a vector in the design space. The set of available designs is restricted by the equality and inequality constraints $h_{m}$, $g_{n}$, as well as by upper and lower bounds for each design variable $x_{p}$. Because of the *duality principle* [20], problems where maximization (or both maximization and minimization) of the objectives is required can be transformed in the form of Equation (1) by sign reversal of the relevant objectives.

In practical cases, it can be assumed that trade-offs exist between any pair of objectives $f_{i}$, $f_{j}$, that is, it is impossible to find a design vector x such that these objectives are simultaneously minimized. The MO approach to the solution of this problem is based on the concept of *Pareto optimality*. Loosely speaking, a point x belongs to the *Pareto-optimal set* (or *Pareto frontier*) if any other solution is worse with respect to at least one of the objectives. This also means that, to find a solution that further improves one of the objectives with respect to x, at least one of the other objectives has to be degraded. No Pareto-optimal solution is better than another, and the choice between two solutions depends on the importance given to each objective for the case at hand. A MO problem is solved when the Pareto set is known.

Classical methods to solve MO problems are typically based on *scalarization*, i.e., a systematic reduction to multiple, single-objective minimization (or maximization) problems. One point of the Pareto set is found for each iteration. In *evolutionary methods*, in contrast, several solutions are computed simultaneously at each iteration. Successive iterations of the algorithms move these solutions towards the Pareto frontier in a process that simulates biological evolution, by selecting solutions based on their fitness to solve the optimization problem at hand.

In this work, we present a semi-analytical approach to the optimization of the geometry of a rectangular MEMS micromirror. While compact analytical models are, of course, ubiquitous in MEMS design, as in any other engineering field, numerical methods (and, prominently, Finite Element Modeling) are typically used for design refinement and, crucially, for systematic optimization. For example, Di Barba and Wiak [16] presented an approach based on a FEM solution of the field distribution (electric or magnetic field) implemented in Comsol Multiphysics, and a genetic algorithm (NSGA-II) for MO. Three different case studies (a comb-finger electrostatic actuator, a torsional magnetic mirror based on a different concept than the one studied in this work, and a Joule thermal actuator) are analyzed. Cobb and Agogino [17] developed a design approach based on case-based reasoning (CBR), an artificial intelligence technique. With this method, past MEMS designs are organized in a hierarchical library from which promising solutions are retrieved based on the specifications of the new design. The retrieved designs are then adapted through parametric optimization and/or multiobjective genetic algorithms (MOGA). The same research group [21] presented a method to reduce the computational burden of optimal design of MEMS based on a hybrid approach, where two different levels of optimization are used: first MOGA is applied to a population of potential designs, then a local optimization on selected solutions is used for further refinement. They use a commercial MEMS accelerometer as a benchmark, and demonstrate improvements on many significant device performances. The optimization of a MEMS accelerometer is also pursued by Pak et al. in [22]. They used an analytical model for the output noise and determined the Pareto frontier with respect to two objectives (the noise spectral density and the area occupancy of the device) with a multiobjective evolutionary algorithm based on decomposition (MOEA/D) [23]. Farnsworth et al. [19] developed a flexible system that applies MO through evolutionary algorithms. The system interfaces with CAD packages or user-provided scripts for device description, and different case studies for either option are presented.

The main advantage of using an analytical model for device description (the approach also used in this work) is that it gives an insightful description of the device behavior, where the value of any desired performance can be easily (and quickly) evaluated. Exploration of the design space is also extremely fast. Moreover, the impact of every design variable on the performance can be evaluated rapidly by direct inspection the analytical expressions, and the robustness of a design with respect to a change in any parameter can be determined analytically. The main disadvantage is, of course, that if one wants to keep the complexity of the model reasonable, more simplifying assumptions are to be made with respect to a full FEM model. As we showed in the above review of the literature, a common approach to decrease the computational burden of FEM in MO is to use efficient MO algorithms to reduce the number of FEM iterations. In this work, we show that reasonably simple analytical models, despite their assumptions, lead to optimal solutions whose performances are within a few percent of the corresponding full FEM model.

The paper is structured as follows: in Section 2, we give a description of the micromirror function and structure, present all the constitutive equations describing its operation, and give a rationale for the choice of the optimization objectives. In Section 3, we present the MO implementation and results. In Section 4, we validate the MO output with FEM.

## 2. Model

In this work, we optimize the geometry of a single-axis magnetic micromirror. Its application is as the vertical (i.e., slow) mirror in a two-mirror system used in a pico-projector. In these systems, the composite light beam coming from the source(s) is reflected by a fast, circular mirror resonating at the line scan frequency, which is of the order of several kilohertz. The oscillating beam is collected by the vertical mirror, which is operated at the much lower video frame frequency. Consequently, the shape of the mirror is rectangular, and the mirror itself works in quasi-static conditions (i.e., at a much lower frequency than that of any mechanical resonance modes).

**Figure 1:**
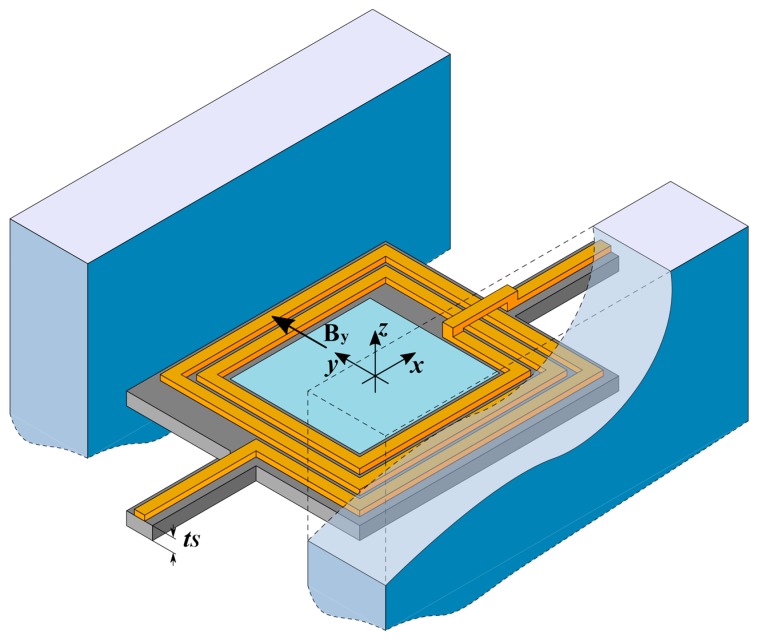
Structure of the mirror assembly with the two permanent magnets (the front magnet is cut away for clarity). The direction of the actuation magnetic field $B_{y}$ and the mirror thickness $t_{S}$ are also shown. The pictured number of loops, $n_{c}$, is 2½.

The structure of the mirror is shown in Figure 1, and all the quantities used in the paper are defined in Table 1. It includes a rectangular plate, at whose center a rectangular reflecting surface (covered with a metal film to increase reflectivity [24]) is positioned. The reflecting region is surrounded by a multi-loop rectangular inductor, in the form of thick metal lines patterned on the plate. Two beams with rectangular cross-section, at two opposite sides of the plate, act as suspending torsional springs for the plate itself, as well as carriers for the electrical input and output lines. Two block-shaped permanent magnets face the other two sides of the plate, and provide the magnetic field required for actuation. When a current $I_{c}$ is forced through the inductor, a Lorentz force appears on the loop sides parallel to the suspensions. The combined effect of these forces results in a torque on the plate, which rotates around the x axis of Figure 1. The restoring torque is provided by the suspension springs. A few simplifying assumptions will be used in the derivation of the constitutive equations. Specifically, we assume that (1) the plate behaves as a rigid body during actuation and consequently (2) all the deformation is sustained by the springs (i.e., we use a lumped parameter model); and (3) the dynamical behavior is dominated by the actuation mode (rotation around x), while other modes are at high frequencies and are not relevant.

### 2.1. Constitutive Equations

A primary performance target for a mirror is, of course, the maximum angular deflection φ. Because the mirror is to be used in quasi-static conditions, static equations can be used to derive φ. First, a linear behavior of the spring is assumed:

(2) $$T_{x}=2k_{\varphi }\varphi$$

where φ is the deflection angle, $T_{x}$ is the external actuation torque, and $k_{\varphi }$ is the spring constant of either of the two suspensions. The value of $k_{\varphi }$ is, in turn:

(3) $$k_{\varphi }=\frac{G_{Si}J_{S\varphi }}{l_{s}},$$

that is, the spring constant is proportional to the shear modulus of silicon $G_{Si}$ and inversely proportional to a geometric factor $J_{S\varphi }$, the torsional constant of the bar cross-section. For rectangular cross-sections, its approximate value (for $w_{s}\geq t_{s}$, a hypothesis to be checked later) is

(4) $$J_{S\varphi }=w_{s}^{3}\;t_{s}\left[\frac{1}{3}-0.21\frac{w_{s}}{t_{s}}\left(1-\frac{w_{s}^{4}}{12\;t_{s}^{4}}\right)\right]$$

An important specification for quasi-static mirrors is also the angular resonance frequency $\omega_{\varphi }$ of the main (torsional) resonance mode. If this frequency is too low, undesired ringing of the angle may occur during actuation. Again, under the assumption of lumped parameter model, this frequency is simply

(5) $$f_{\varphi }=\frac{1}{2\pi }\sqrt{\frac{2\;k_{\varphi }}{J_{Px}}}$$

with $J_{Px}$ being the moment of inertia of the plate along the x axis. The moment of inertia is the sum of the moments of inertia of the silicon plate $J_{Sx}$ and of the coils $J_{Cx}$. For simplicity, we assume negligible thickness for both. Moreover, we also assume that the mass of the metal lines is uniformly distributed on the silicon surface, with the exclusion of the central reflecting area. If we think of the coils as a uniform rectangular thin plate with a rectangular hole at its center, it is easy to show that this equivalent plate has a moment of inertia

(6) $$J_{Cx}=\frac{1}{12}M_{c}\frac{l_{x}l_{y}^{3}-d_{x}d_{y}^{3}}{l_{x}l_{y}-d_{x}d_{y}},$$

where $l_{x}$, $l_{y}$, $d_{x}$, $d_{y}$ are the plate and hole sides, respectively, and $M_{c}$ is the total coil mass. This mass is

(7) $$M_{c}=\mu_{l}t_{l}w_{l}l_{c},$$

where $\mu_{l}$ is the density of the metal (which we assumed to be aluminum), $t_{l}$ its thickness, and $l_{c}$ the total length of the coil. This length can be computed by summing the length of each individual coil segment, obtaining a closed form for the sum:

(8) $$l_{c}=2n_{c}\left(d_{x}+d_{y}+\left(2n_{c}-1\right)p_{l}+2w_{l}\right).$$

The total moment of inertia is then:

(9) $$J_{Px}=\frac{1}{12}\mu_{Si}t_{l}l_{x}l_{y}^{3}+J_{Cx},$$

$\mu_{Si}$ being the density of the mirror material (which we assumed to be silicon).

**Figure 2:**
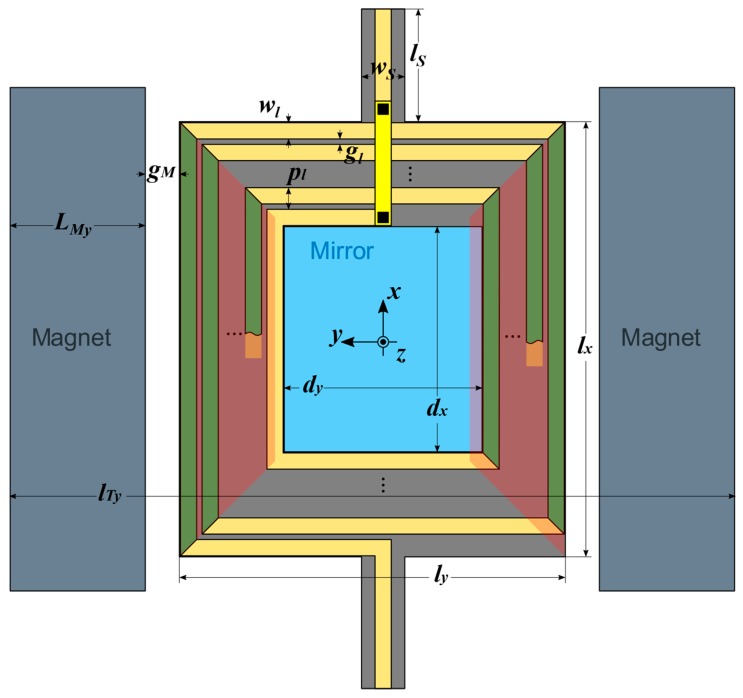
Top view of the mirror. Actuation coil segments are in green, areas for the calculation of the equivalent magnetic field are in red.

In addition to the mechanical Equations (2)–(9), a few more equations are necessary to define the geometry of the mirror. Their interpretation, with the aid of Figure 2 and Table 1, is straightforward, and so they are given below without further comment:

(10) $$p_{l}=g_{l}+w_{l},$$

(11) $$l_{x}=\left(2\;n_{c}+1\right)p_{l}+d_{x},$$

(12) $$l_{y}=\left(2\;n_{c}+1\right)p_{l}+d_{y},$$

(13) $$l_{Ty}=2\;g_{m}+2\;l_{My}+l_{y}.$$

Finally, we need an expression for the electromagnetic torque $T_{x}$ exerted on the plate because of the Lorentz force of the external magnetic field B. In general, the magnetic field distribution caused by the magnets has non-null components along the three axes. Consequently, torques along all three directions act on the plate. However, we assume that rotations around y and z are rejected by the high associated torsional stiffnesses. For rotations around x, and at least for small angles, the only effective field component is $B_{y}$. This component creates a Lorentz force only on the coil segments along x (the ones in green color in Figure 2). The generic expression for the torque would then be:

(14) $$T_{x}=\underset{k}{\sum }T_{x,k}=I_{c}\underset{k}{\sum }a_{k}\overset{}{\underset{\Lambda_{k}}{\int }}\;\;B_{y}\left(r\right)dr$$

where the sum is extended over each coil segment, $a_{k}$ is the distance of the *k*th segment from the plate center (i.e., the elemental torque lever arm), and the integral is a line integral along the *k*th segment length. The use of Equation (14) implies knowledge of the field distribution over the plate. In this paper, we assume that the magnets are block-shaped (parallelepipedal), a geometry for which a closed expression for the magnetic field is available [25]. Even with this closed form, however, the integrals in Equation (14) would be very cumbersome to evaluate for a generic geometry, and subsequent FEM simulations (see Section 4) will show that this level of model detail is not really required for accuracy. We then simply assume that $B_{y}$ is constant over all the segments, and choose as this constant value the value of $B_{y}$ at the center of the trapezoid shaded in red in Figure 2, for which a closed expression can be written (see Appendix A). Equation (14) then reduces to:

(15) $$T_{x}=I_{c}B_{y}\underset{k}{\sum }a_{k}s_{k}$$

where $s_{k}$ is the length of the *k*th segment. The sum in Equation (15) has the dimensions of an area and can be interpreted as an equivalent area $A_{c}$ of the coil. A cumbersome derivation gives a closed expression for $A_{c}$:

(16) $$A_{c}=\frac{1}{12}\;n_{c}\;(12\;d_{x}\;\left(\left(n_{c}-1\right)p_{l}+w_{l}+d_{y}\right)+6\;d_{y}\;\left(\left(2\;n_{c}-1\right)p_{l}+2\;w_{l}\right)+\;24\;n_{c}\;p_{l}\;w_{l}+16\;n_{c}^{2}\;p_{l}^{2}-18\;n_{c}\;p_{l}^{2}-18\;p_{l}\;w_{l}+5\;p_{l}^{2}+12\;w_{l}^{2}).$$

Remarkably, Equations (2)–(16) and (A2) can be solved analytically for most of the interesting design objectives, which can thus be expressed as a function of several potential design variables (the number of loops $n_{c}$, the spring length $l_{s}$, etc.), and a few constants, such as the width of the reflector $d_{y}$ (which is imposed by the application) or the gap $g_{m}$ between the mirror and the magnets (which is imposed by the technology). For example, the expression for the total width $l_{Ty}$ is

(17) $$l_{Ty}=d_{y}+2g_{m}+2l_{My}+\left(1+2n_{c}\right)\left(g_{l}+\;w_{l}\right).$$

Comparatively more complex expressions, for the current $I_{c}$, the resonance frequency $\omega_{\varphi \;}$ and other quantities exist, though they are not reported here for brevity. All these expressions are extracted from the above equations with the aid of an algebraic manipulation software package. Consequently, we can write closed analytical form for the desired optimization objectives $f_{k}\left(x\right)$ as a function of the design variables.

### 2.2. Choice of the Optimization Objectives

To state the mirror optimization problem into the form of Equation (1), a suitable choice of the objectives is required. The maximum angular deflection of the mirror seems as the obvious first choice. In practical cases, however, the maximum angle is typically dictated by the application, and thus not directly subject to maximization. Moreover, the maximum angle is directly proportional to the current $I_{c}$ through Equations (2) and (15), so that the real objective is the minimization is the current itself. We then choose $I_{c}$ as the first objective function.

Another figure of merit for micromirrors is the total size, which is especially important for pico-projectors used in mobile applications. The correct choice of an objective related to size is dependent on the specifics of the applications, but a reasonable choice is the minimization of the total width $l_{Ty}$, which depends both on the size of the plate and the size of the magnets. There is a clear trade-off between the minimization of $I_{c}$ and $l_{Ty}$, because to keep the deflection angle constant at smaller currents, the design requires a larger number of loops and/or larger magnets, and both increase the mirror size.

Another attractive objective is the maximization of the torsional resonance frequency $f_{\varphi }$. The actuation frequency for vertical scanning in pico-projectors equals the video frame rate, which is typically around 50 Hz. A resonance frequency much higher than this value is thus required to minimize mechanical ringing during actuation.

### 2.3. Choice of the Design Variables

The choice of the most interesting potential design variables is not necessarily trivial. If all or most unknowns were used as one of the design variables $x_{p}$, the complexity of the problem would be unnecessarily high. Many of the unknows are set by the application (this is the case, for example, of $d_{x}$ and $d_{y}$), or their value can be easily assigned (the case of $g_{m}$), as there is no trade-off between objectives consequent to this choice. For example, we set the aforementioned distance $g_{m}$ between the mirror and the magnets at its minimum value compatible with fabrication constraints, because both the current $I_{c}$ and the width $l_{Ty}$ decrease as $g_{m}$ decreases, and the resonance frequency is not a function of $g_{m}$. To reduce complexity, we also chose to set some variables, such as the length $l_{Mx}$ and height $l_{My}$ of the magnets, to technically sound values. For other variables, such as the spring width $w_{s}$ and plate thickness $t_{s}$, an analysis of the closed expressions for each of the objectives showed that the current $I_{c}$ was an increasing function of both $w_{s}$ and $t_{s}$, as confirmed by physical intuition, while $l_{Ty}$ was obviously independent of both. Despite a similar analysis showing that $\omega_{\varphi }$ increases with both $w_{s}$ and $t_{s}$, preliminary tests showed that the objective of a high resonance frequency is the less constraining of the three. Consequently, we set $w_{s}$ and $t_{s}$ at the smallest value compatible with reasonable technological constraints, which also ensures that the constraint $w_{s}\geq t_{s}$, required by Equation (4), is fulfilled. The values for each of the parameters are shown in Table 1.

## 3. MO Implementation and Results

After this complexity reduction, we remain with four design variables, three of them ($l_{s}$, $w_{l}$ and $n_{c}$) referring to the mirror geometry, and one ($l_{My}$) to the magnet geometry. We then have all the components required to state the MO problem in closed form:

(18) $$\left\{ \begin{array}{l} \mathrm{minimize}\;I_{c}(\;l_{s},w_{l},l_{My},n_{c}),\;L_{Ty}(\;l_{s},w_{l},l_{My},n_{c}),-f_{\varphi }(\;l_{s},w_{l},l_{My},n_{c})\\ \mathrm{constrained}\;\mathrm{to}\\ \;\;\mathrm{Equations}\;(2)–(13),(15)–(16),(A2)\\ \;\;l_{sL}\leq l_{s}\leq l_{sH},\\ \;\;w_{lL}\leq w_{l}\leq w_{lH},\\ \;\;l_{MyL}\leq \;l_{My}\leq l_{MyH},\\ \;\;n_{cL}\leq n_{c}\leq n_{cH}. \end{array}\right.$$

The range limits for each design variable are given in Table 2. Because the focus of the approach presented in this work is not on the MO algorithm in itself, but rather on the choice of a reduced analytical model for the objective and constraint functions in the context of MO, we implemented a simple, classical MO algorithm, the ε-constraint method, that first appeared in [26].

Despite its simplicity, it allows reconstruction of the Pareto-optimal set also for non-convex domains, a case where other classical methods fail. Briefly, in the *ε*-constraint method, all objectives but one are restricted below a specific parameter $\varepsilon_{k}$, and the remaining objective is minimized:

(19) $$\{\begin{array}{ll} \mathrm{minimize}f_{\kappa }\left(x\right),\;\; & \\ \mathrm{constrained}\;\mathrm{to} & \\ f_{k}\left(x\right)\leq \varepsilon_{k}, & k=1,\ldots ,K,\;k\neq \kappa \\ h_{m}\left(x\right)=0, & m=1,\ldots ,M\\ g_{n}\left(x\right)\geq 0, & n=1,\ldots ,N\\ x_{pL}\leq x_{p}\leq x_{pH}, & p=1,\ldots ,P. \end{array}$$

To avoid problems in numerical accuracy, the objectives were normalized to lie in the range [0, 1] through the transformation:

(20) $${\tilde{f}}_{k}=\frac{f_{k}-f_{k,I}}{f_{k,N}-f_{k,I}}$$

where $f_{k,I}$ and $f_{k,N}$ are the elements of the ideal and nadir vector, respectively, representing the lower and upper bounds for each objective over the design space. For each *k*, the value of $f_{k,I}$ is determined by minimization of the *k*th objective alone, disregarding the requirement on the other objectives. The nadir vector was estimated by using the *payoff method* [27], which assigns as the upper bound of one objective the maximum assumed by that objective at the points that give the ideal (lower bound) value for all other objectives.

**Figure 3:**
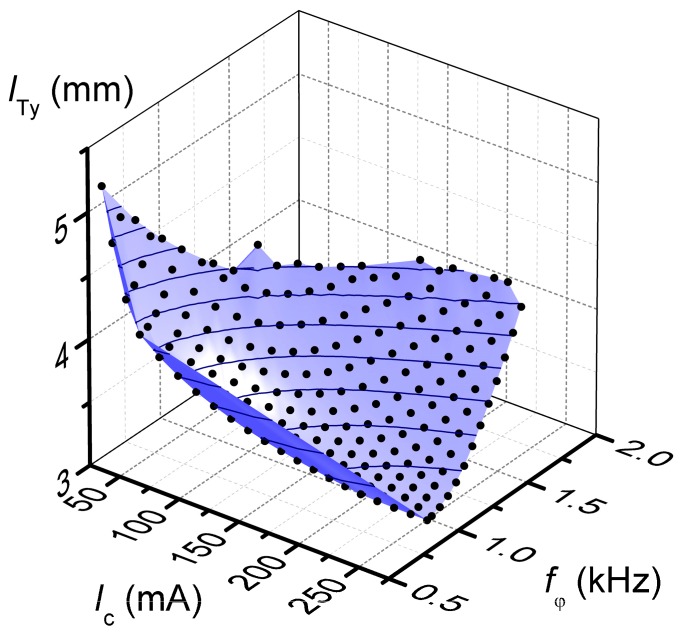
Interpolated Pareto frontier for the mirror problem, as determined by numerical solution of (17), with curves at constant $l_{Ty}$ plotted over the surface. Each black dot is a Pareto-optimal solution lying (by definition) on the Pareto frontier.

The solution of the minimization problem (19) was implemented as a Wolfram Mathematica script. Specifically, $l_{Ty}$ was minimized numerically, with the constraint parameters $\varepsilon_{Ic}$ and $\varepsilon_{\omega \varphi }$ swept uniformly between 0 and 1, for a total of more than 400 minimizations. To exclude technically unrealistic solutions, an inequality constraint to require a resonance frequency above 500 Hz (one order of magnitude above typical scanning frequencies), as well as an upper limit of 250 mA for the current, were also included. The total time required to determine the Pareto frontier was about 1.6 s for each point on a PC with 8 GB of RAM. Because each design variable is optimized over the reals, non-manufacturable values for $n_{c}$, which must be in the form p+1/2, with integer p, may result. The impact of this issue will be verified with the FEM simulations. The optimal solutions in the objective space, defining the Pareto frontier, are shown in Figure 3.

## 4. FEM Validation

The algorithm guarantees that the solutions lie on the Pareto frontier. However, the use of a reduced analytical model means that a slightly different problem than the one including the full physics of the actual mirror is solved. To evaluate the impact of this simplification, we verified via FEM simulation in ANSYS each of the optimal geometries calculated with the procedure described in Section 3.

We performed both static and modal FEM analyses. Static simulations were used to determine the difference between the nominal deflection angle (10°) and the actual angular deflection at the nominal current obtained by the MO. Modal analysis was instead used to verify if the lowest resonance frequency was really the one of the main torsional mode, and to evaluate the difference between the corresponding FEM frequency and the frequency obtained by the MO. For FEM simulations, the value of $n_{c}$ from the MO was rounded to the closest number of the form p+1/2, with integer p. This choice ensured that the FEM design was compatible with the structure shown in Figure 1 and Figure 2, while also introducing a systematic error that will be evaluated below. The geometry was meshed with the SOLID185 structural element, with ten elements along the z direction. The center of the plate was meshed with a material with the properties of (100) silicon, while the outer area, i.e., the one carrying the coil, was meshed with an equivalent material with the same elastic constants of silicon, but with an equivalent mass density matched to distribute the total mass of the coil, as determined by Equation (7), over the whole surface. The mesh was refined at the connections between springs and plate, where a concentration of the stress is expected. The number of elements for the simulation of a single geometry, though dependent on the values of the design parameters, was typically around 125,000. To verify proper convergence of the FEM results, a selected number of geometries was simulated with a finer mesh of around 400,000 elements; the calculated deflection angles and resonance frequencies differed, on average, for less than 0.8% with those of the coarser mesh.

**Figure 4:**
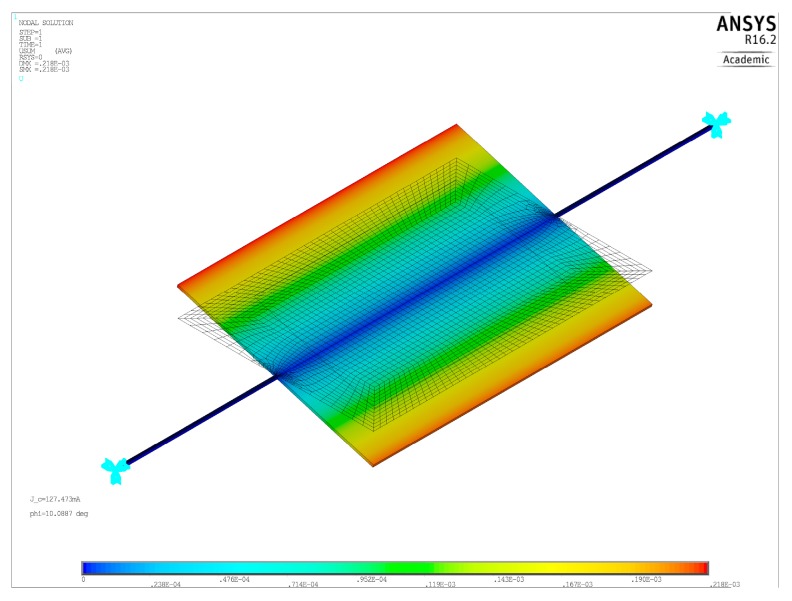
Finite Element (FEM) plot of the static deflection for a sample geometry.

In the static FEM simulations, to include the effect of the non-uniform magnetic field, a distributed vertical force (i.e., a pressure) was applied in the active areas of the plate (the ones shaded in red in Figure 2) in the ANSYS script. The value of this pressure was dependent on the magnetic field. Specifically, we assumed a uniform surface current density $\sigma_{c}=n_{c}I_{C}/\left(\left(l_{y}-d_{y}\right)/2\right)$ (Am^−1^) flowing in the active areas. We then applied at each mesh node of the active area a pressure equal to $\sigma_{c}B_{y}\left(x,y\right)$, where (x,y) were the coordinates of the node, and the expression of $B_{y}$ was determined through the formulas in Appendix A. To include the effect of large rotations of the plate, which produces a reduction of the effective arm of the magnetic torque with respect to the rest position, the pressure was further reduced by a factor cosφ, with φ being the target angle (10°). A typical deflected shape for static simulations is shown in Figure 4. For modal FEM simulations, the frequency of the lowest mode was computed, and it was verified that it corresponded to the torsional mode.

**Figure 5:**
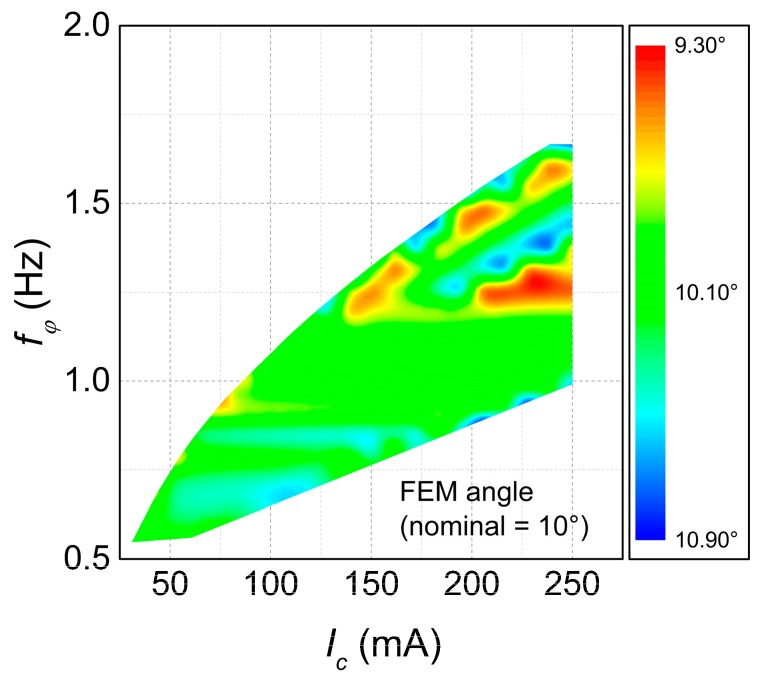
FEM angular deflection of the optimal mirrors (linear case).

Overall, there was a good agreement between the performances of the geometries as optimized by the Mathematica code, both for the static deflection and resonance frequency. Over the 271 geometries of Figure 3, the average FEM angle was 10.15°, or only about 1.5% larger than the nominal value of 10°, and the standard deviation was 0.33°. Interestingly, most of this dispersion can be attributed to the rounding of the number of loops $n_{c}$. Indeed, there is a high correlation ($R^{2}=0.919$) between the FEM angle and the rounding error of $n_{c}$ and, on average, a rounding of half a coil creates a difference of 0.60° in the deflection. The actual FEM angle, projected on the $I_{C}-f_{\varphi }$ plane, is shown in Figure 5.

To verify the impact of non-linearity, the same geometries were also simulated with geometric non-linearity active in the ANSYS script. The impact of non-linearity is small, with an average deflection of 10.09°, or 0.88% larger than the nominal value, and a standard deviation of the angle of 0.32°.

**Figure 6:**
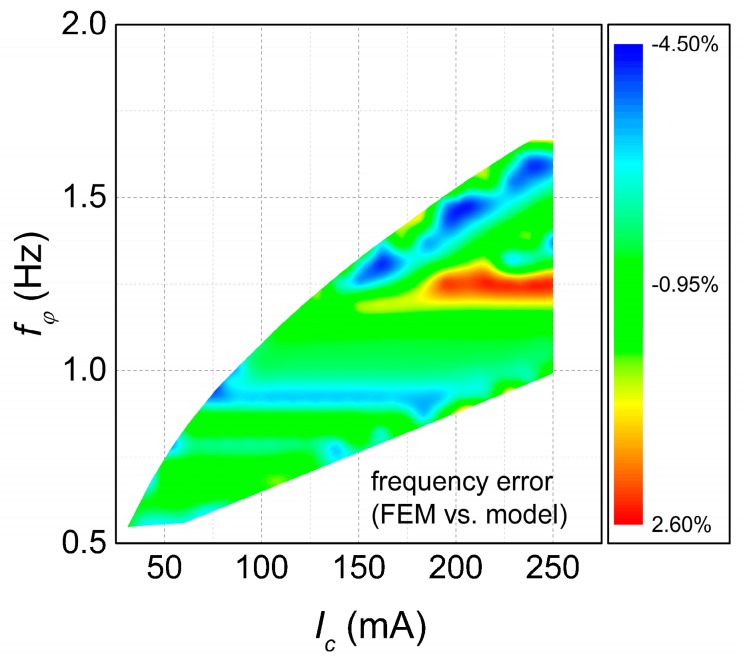
Relative error for the resonance frequency between the FEM case and the MO (Multiobjective Optimization) case.

Comparable agreement existed also for the resonance frequency, with an average relative error between the FEM value and the MO value of around 1.22% and a standard deviation of 1.48%. While in this case the correlation is less clear, a part of this dispersion can be also attributed to the rounding of $n_{c}$. The relative error on the resonance frequency between the FEM and the MO value is shown in Figure 6.

## 5. Conclusions

In this work, we propose a fast method for the optimization of a MOEMS micromirror, based on an analytical description of the mirror constitutive equations. Because of the existence of closed-form expressions for any design objectives, the impact of a change in any design variables can be theoretically evaluated. Three important design objectives (the driving current, the micromirror size, and the first resonance frequency) are then chosen, and the optimization is carried out in the MO sense, that is, by exploration of the design space to determine the structure of the Pareto frontier. Because of the choice of the descriptive model for the mirror, only a few minutes on a standard PC are enough to obtain the Pareto frontier, even using a simple optimization algorithm. FEM validation of the Pareto-optimal solutions shows that actual performances of the device are within a few percent of those determined with the proposed method. The main advantage of this approach is the speed with which several Pareto-optimal solutions can be determined. It is also worth mentioning that in our case a closed solution of the constitutive equations of the system exists, but the method can be applied even in cases where this solution has to be found numerically, though with a reduction in speed.

While the accuracy of this solutions can be sufficient for the actual design, as shown by our results, they could also be used as starting points for a subsequent multiobjective optimization with efficient MO algorithms coupled with FEM simulations, reducing the number of iterations and thus limiting greatly the accurate but time-consuming FEM evaluations.

## Figures and Tables

**Table 1:** List of symbols used in the text, with their assigned values where relevant.

Symbol	Unit	Definition	Value
$A_{c}$	m^2^	equivalent area of coil (are such that $T_{x}=I_{c}B_{y}A_{c}$)	-
$a_{k}$	mm	distance of *k*th active coil segment from plate center	-
*B_R_*	T	magnetic material remanence	1.4
$B_{y}$	T	magnetic field along y at the center of active area (Figure 2)	-
$d_{x}$	mm	length of the reflective surface	2.5
$d_{y}$	mm	width of the reflective surface	1.5
$f_{\varphi }$	Hz	angular resonance frequency of main torsional mode	-
*g_l_*	μm	distance (gap) between metal lines	10
$g_{m}$	mm	distance (gap) between the mirror and the magnets	
$G_{Si}$	GPa	shear modulus of silicon along x	79.5
$I_{c}$	mA	coil current	-
$J_{Px}$	kg·m^2^	moment of inertia of the plate	-
$J_{S\varphi }$	m^4^	torsional constant of the spring cross-section	-
$k_{\varphi }$	N·m	torsional spring constant of one spring along φ	-
$l_{c}$	mm	Total length of the coil	-
$l_{My}$	mm	magnet width along y	-
$l_{M\left\{x,z\right\}}$	mm	magnet dimensions along {x,z}	{5, 5}
$l_{s}$	m	spring length	-
$l_{Ty}$	mm	total assembly width (mirror + magnets)	-
$l_{x}$	m	length of mirror plate (including room for the coil)	-
$l_{y}$	m	width of mirror plate (including room for the coil)	-
$M_{c}$	kg	total mass of the coil	-
$n_{c}$	-	number of coils (counting also half-coils)	-
$p_{l}$	μm	metal line pitch	-
$s_{k}$	mm	length of *k*th active coil segment	-
$t_{l}$	μm	Metal thickness	10
$t_{s}$	μm	thickness of plate and springs	30
$T_{x}$	N·m	torque on the plate	-
$w_{l}$	μm	metal line width	-
$w_{s}$	μm	spring width	30
φ		rotation along main axis (*x* axis)	10
$\mu_{l}$	kg/m^3^	aluminum density	2700
$\mu_{Si}$	kg/m^3^	silicon density	2330

**Table 2:** Minimum and maximum allowed values for the design variables.

Variable	Max	Min
$l_{s}$	0.2 mm	2 mm
$w_{l}$	20 μm	200 μm
$n_{c}$	1.5	199.5
$l_{My}$	0.3 mm	1 mm

## Appendix A

Consider a uniformly magnetized parallelepiped magnet, centered at the origin, with sides $l_{Mx}$, $l_{My}$, $\;l_{Mz}$, and residual flux density (remanence) $B_{R}$. The magnetic field component $B_{y}$ along the y direction, at a distance y from the magnet center, is [25]

(A1) $$B_{y}^{\prime }\left(0,y,0\right)=\frac{B_{R}}{\pi }\;\left[\arctan\left(\frac{\frac{l_{Mx}\;l_{Mz}}{4}\;}{\;\left(y-\frac{l_{My}}{2}\right)\;\sqrt{\frac{l_{Mx}^{2}}{4}+({\left(y-\frac{l_{My}}{2}\right)}^{2}+\frac{l_{Mz}^{2}}{4}}}\right)-\arctan\left(\frac{\frac{l_{Mx}\;l_{Mz}}{4}\;}{\;\left(\frac{l_{My}}{2}+y\right)\;\sqrt{\frac{l_{Mx}^{2}}{4}+{\left(\frac{l_{My}}{2}+y\right)}^{2}+\frac{l_{Mz}^{2}}{4}}}\right)\right].$$

Consequently, the field at a distance y from the origin generated by two identical magnets, placed as shown in Figure 2, is

(A2) $$\begin{array}{l} B_{y}=B_{y}^{\prime }\left(0,g_{m}+\frac{l_{My}}{2}+\frac{l_{y}}{2}+y,0\right)+B_{y}^{\prime }\left(0,-g_{m}-\frac{l_{My}}{2}-\frac{l_{y}}{2}+y,0\right)=\\ =\frac{B_{R}}{\pi }\{\arctan\left[\frac{\frac{l_{Mx}\;l_{Mz}}{4}}{\;\left(g_{m}+\frac{l_{y}}{2}+y\right)\sqrt{\frac{l_{Mx}^{2}}{4}+{\left(g_{m}+\frac{l_{y}}{2}+y\right)}^{2}+\frac{l_{Mz}^{2}}{4}}}\right]-\;\arctan\left[\frac{\frac{l_{Mx}\;l_{Mz}}{4}}{\;\left(g_{m}+l_{My}+\frac{l_{y}}{2}+y\right)\sqrt{\frac{l_{Mx}^{2}}{4}+{\left(g_{m}+l_{My}+\frac{l_{y}}{2}+y\right)}^{2}+\frac{l_{Mz}^{2}}{4}}}\right]\\ +\arctan\left[\frac{\frac{l_{Mx}\;l_{Mz}}{4}}{\;\left(-g_{m}-l_{My}-\frac{l_{y}}{2}+y\right)\sqrt{\frac{l_{Mx}^{2}}{4}+{\left(-g_{m}-l_{My}-\frac{l_{y}}{2}+y\right)}^{2}+\frac{l_{Mz}^{2}}{4}}}\right]-\arctan\left[\frac{\frac{l_{Mx}\;l_{Mz}}{4}}{\;\left(-g_{m}-\frac{l_{y}}{2}+y\right)\sqrt{\frac{l_{Mx}^{2}}{4}+{\left(-g_{m}-\frac{l_{y}}{2}+y\right)}^{2}+\frac{l_{Mz}^{2}}{4}}}\right]\} \end{array}$$

The field at the center of the active area can be found from Equation (A2) by the substitution $y=d_{y}/2+\left(l_{y}-d_{y}\right)/4$.
